# The role of major allergens Art v 1 and Art v 3 in *Artemisia* pollen-induced asthma: a mouse model study

**DOI:** 10.3389/fimmu.2025.1590791

**Published:** 2025-06-03

**Authors:** Nazugum Akhtemova, Akbota Sergazina, Turlan Bolatbekov, Guliza Rakhmatullayeva, Elmira Tailakova, Kairat Tabynov, Gleb Fomin, Lan Zhao, Zhongshan Gao, Kaissar Tabynov

**Affiliations:** ^1^ International Center for Vaccinology, Kazakh National Agrarian Research University (KazNARU), Almaty, Kazakhstan; ^2^ Preclinical Research Laboratory with Vivarium, M. Aikimbayev National Research Center for Especially Dangerous Infections, Almaty, Kazakhstan; ^3^ T&TvaX LLC, Almaty, Kazakhstan; ^4^ College of Agriculture and Biotechnology, Zhejiang University, Hangzhou, China; ^5^ Institute of Immunology, School of Medicine, Zhejiang University, Hangzhou, China; ^6^ Department of Allergy, Beijing Shijitan Hospital, Capital Medical University, Beijing, China; ^7^ Republican Allergy Center, Research Institute of Cardiology and Internal Medicine, Almaty, Kazakhstan

**Keywords:** *Artemisia pollen*, Art v 1, Art v 3, allergic asthma, mouse model, pulmonary pathology

## Abstract

**Background:**

*Artemisia* pollen is a major airborne allergen contributing to seasonal allergic rhinitis and bronchial asthma worldwide. However, the specific allergenic potential of different *Artemisia* species and their major allergens, Art v 1 and Art v 3, remains poorly understood.

**Methods:**

This study utilized a BALB/c mouse model to comparatively assess the allergenic potential of *A. vulgaris, A. absinthium*, and *A. annua.* Mice were sensitized and challenged with standardized pollen extracts, and allergic responses were evaluated through serum IgE levels, airway hyperreactivity, ear swelling, and histopathological lung analysis. The correlation between allergen content (Art v 1 and Art v 3) and allergic outcomes was also examined.

**Results:**

*A. vulgaris* exhibited the highest allergenicity, inducing the most pronounced IgE response, airway hyperresponsiveness, and severe pulmonary pathology. *A. absinthium* displayed intermediate allergenic potential, while *A. annua* elicited the mildest allergic response. Art v 1 levels strongly correlated with key clinical and pathological markers of asthma, whereas Art v 3 showed no significant association. Notably, the Art v 1/Art v 3 ratio emerged as the strongest predictor of pulmonary pathology.

**Conclusion:**

These findings establish Art v 1 as the key driver of *Artemisia*-induced allergic inflammation and emphasize its dominance over Art v 3 in pollen as a critical factor in the development of allergic lung pathology. These findings provide new insights into the molecular basis of *Artemisia* pollen allergy and may inform future diagnostic and therapeutic strategies.

## Introduction

Seasonal allergic rhinitis, or pollinosis, caused by plant pollen, is a growing global health concern, affecting up to 30% of the population (1–3). Among allergenic plants, the genus *Artemisia* (mugwort) stands out as a major contributor, with its pollen ranked among the top ten worldwide (4, 5). Belonging to the *Asteraceae* family, *Artemisia* encompasses ~400 species adapted to diverse environments on every continent except Antarctica (6). The genus demonstrates the highest species diversity in Central Asia, Russia, Mongolia, China, Europe, and North America (7–9), marking these regions as ecological hotspots and reinforcing Artemisia’s relevance in allergenic research.

Their extended flowering periods contribute significantly to seasonal allergic reactions in the local population (10), reflecting global patterns where *Artemisia* pollen is recognized as a primary trigger for respiratory diseases, including asthma - the most severe manifestation of allergies (11). Studies indicate that nearly 50% of pollen-allergic patients develop seasonal asthma within nine years, while even those without overt asthma often exhibit bronchial hyperresponsiveness during pollen seasons (12, 13). Pollen-induced asthma not only impairs respiratory function but also causes chronic tissue damage, leading to a marked decline in quality of life for affected individuals (14).

Compounding the challenge, cross-reactive responses frequently complicate allergic reactions to *Artemisia* pollen. Sensitized patients often experience allergic responses not only to other members of the *Asteraceae* family but also to structurally similar foods such as celery, carrots, and certain spices (15). These cross-reactions intensify the complexity of allergy management, highlighting the critical need to identify key allergens and understand their interactions to improve diagnostic and therapeutic strategies.

Ten allergens from *Artemisia* pollen have been identified and are listed in the International Union of Immunological Societies (IUIS) allergen nomenclature database (16). Two major proteins, Art v 1 and Art v 3, have been identified as the major allergens in *Artemisia* pollen, playing a critical role in the allergy immune response (17). Art v 1 is a defensin-like protein and the primary trigger of IgE-mediated immune responses, making it a key factor in allergic inflammation (18, 19). Art v 3, a lipid transfer protein (LTP), differs from Art v 1 in its ability to induce localized and systemic allergic reactions, thereby increasing the risk of severe respiratory inflammation and lung tissue damage (20). Sensitization to both allergens has been associated with an increased risk of asthma (18, 20).

While previous studies, such as Pablos et al. (21), have compared *Artemisia* species based on allergen content, including Art v 1 and its homologues, this study is the first to employ a laboratory mouse model to directly compare the allergenic potential of *Artemisia vulgaris* (common mugwort), *A. annua* (annual wormwood), and *A. absinthium* (sweet wormwood), which are widely distributed across various geographic regions, including Central Asia, Europe, North America, and East Asia (10). In this assessment, we specifically examined the allergenic potential of these species in relation to the content of the most clinically significant major allergens, Art v 1 and Art v 3, in pollen extracts, including their quantitative ratio. By analyzing both absolute concentrations and relative proportions of these key allergens, we aimed to determine how their composition influences the allergenicity of different *Artemisia* species. By identifying the most allergenic species among those studied and demonstrating a strong correlation between the Art v 1/Art v 3 ratio and clinical allergy symptoms, this research introduces a novel parameter for predicting allergenic risk.

## Material and methods

### Allergen preparation and major protein quantification

Native pollen extracts of *A. vulgaris*, *A. absinthium*, and *A. annua* were generously supplied by the manufacturer Burly (Almaty, Kazakhstan). The extracts, sourced from batch 190823 (**Table 1**), were provided at an initial concentration of total protein 3.11-3.68 mg/mL (10,000 PNU/mL). The quantitative content of the major proteins Art v 1 and Art v 3 in *Artemisia* extract samples was determined using a previously described method (18). To evaluate the overall protein composition and ensure comparability between extracts, SDS-PAGE was performed under reducing conditions using a 12% polyacrylamide gel, followed by Coomassie Brilliant Blue staining (**Supplementary Figure S1**). To facilitate sensitization studies, bulk preparations of native *Artemisia* pollen extracts were adsorbed onto aluminum hydroxide (Al^3+^ 10 mg/ml, InvivoGen, San Diego, CA, USA) at a 50:50 volume ratio. The resulting allergen formulations were stored at 2–8°C under controlled conditions until needed for experimentation.

**Table 1:** Major allergen content in pollen extracts of different *Artemisia* species.

Pollen extracts	*A. vulgaris*	*A. absinthium*	*A. annua*
Art v 1 homologues (μg allergen/mg pollen protein)	25.30	12.49	15.81
Art v 3 homologues (μg allergen/mg pollen protein)	15.19	7.59	57.59
Art v 1 (μg/ml)	93.1	38.84	55.96
Art v 3 (μg/ml)	55.8	23.6	193.1
Ratio of Art v 1 to Art v 3	1.66	1.64	0.28
Total protein in pollen extract (mg/ml)	3.68	3.11	3.54

### Mouse sensitization and allergy provocation

Our murine model effectively replicates primary sensitization through intraperitoneal (i.p.) allergen exposure, followed by a challenge phase that simulates the natural boosting of primary sensitization seen in allergic patients, as previously described in established sensitization protocols (22, 23). If briefly, 8- to 12-week-old pathogen-free (SPF) male BALB/c mice (n=5/group, 20 mice in total) were randomly assigned to four groups. Mice were injected i.p. twice at 14-day intervals with total protein 350 µg/200 µL of *A. vulgaris*, *A. absinthium*, and *A. annua* extracts (Burly, Almaty, Kazakhstan) sorbed on aluminum hydroxide (Al^3+^ 1 mg/mouse). Negative control mice (n=5) were similarly injected with PBS (200 μL). On day 21, all mice were subjected to three times challenge at daily intervals (on days 21, 23, 25) by inhalation of *Artemisia* pollen extracts (total protein 350 µg/group) according to the previously described method (23), as well as intranasal injection under ketamine-xylazine anesthesia of allergen at a dose of total protein 70 µg/20 μl or the same volume of PBS (negative control). On day 27, all mice were assessed for airway responsiveness after methacholine or PBS inhalation. On the final day of the experiment (day 28), all mice underwent an ear swelling test, had blood samples taken to determine total, allergen-specific IgE levels, and were necropsied for histological lung analysis to assess inflammatory reactions.

### Determination of total IgE by ELISA

Total IgE antibodies were quantified using the ELISA MAX™ Standard Set Mouse IgE (BioLegend, San Diego, CA, USA) according to the manufacturer’s protocol, with results reported in µg/mL.

### Allergen-specific IgE quantification by ELISA

96-well plates (Thermo Fisher Scientific, Waltham, MA, USA) were coated with 100 μL per well of capture antibody specific for IgE (from the ELISA MAX™ Standard Set Mouse IgE, BioLegend) and incubated overnight at 2–8°C. The following day, the plates were blocked with 200 μL per well of blocking buffer and incubated at room temperature (RT) for 1 h on a PST-60HL thermal shaker (BIOSAN, Latvia). After blocking, the plates were washed four times with wash buffer. Mouse serum samples, diluted 1:10 in assay diluent, were added at 100 μL per well and incubated at RT for 2 h with shaking, followed by four washes. Subsequently, 100 μL per well of biotinylated allergen extracts from *Artemisia vulgaris*, *A. absinthium*, and *A. annua* were added and incubated at RT for 1 h with shaking. Biotinylation of allergens was performed using the EZ-Link™ NHS-Biotin kit (Thermo Fisher Scientific, Rockford, Illinois, USA) according to the manufacturer’s instructions, ensuring selective labeling of primary amine groups while preserving allergenic properties. The plates were then washed five times, followed by the addition of 100 μL per well of TMB substrate (BioLegend, USA) at RT for 15 min to initiate the colorimetric reaction. The reaction was stopped by adding 100 μL per well of 2.5 M H_2_SO_4_, and absorbance was measured at 450 nm using a Chromate 4300 microplate reader (Awareness Technologies, USA).

### Ear swelling test

All mice received an intradermal injection of 10 µL of pollen extracts from *A. vulgaris*, *A. absinthium*, and *A. annua* (total protein 35 µg/mouse) into the right auricle, while the negative control group was injected with PBS. After 1.5 to 2 h, auricle thickness was measured using an electronic digital micrometer (MCC-25 DSWQ0-100II, China). The results are expressed as the difference in thickness between the right auricle (allergen-injected) and the left auricle (PBS-injected) in mm.

### Lung hyperresponsiveness assessment

Airway reactivity to methacholine was evaluated using a whole-body plethysmography (WBP-M) system (Shanghai TOW Intelligent Technology Co. Ltd., Shanghai, China), following the method described by Hamelmann et al. (19). All mice were placed in the plethysmography chamber and exposed to aerosolized methacholine (25 mg/mL) or PBS (negative control) inhalation for 5 min. Airway resistance was measured and expressed as enhanced pause (Penh), an indicator of airway obstruction. The response to methacholine was assessed by calculating Penh values using ResMass 1.4.2 software (TOW, China).

### Lung histology analysis

Mouse lungs were fixed in 10% buffered formaldehyde, rinsed with water, and processed through a series of dehydration and clearing steps, including four washes with 100% isopropyl alcohol and two washes with xylene. The tissues were then infiltrated with paraffin in four stages to create paraffin blocks, which were subsequently sectioned into 5-µm slices using a microprocessor-controlled microtome (MZP-01, KB Technom, Russia).

The tissue sections were deparaffinized with two washes of xylene, followed by rehydration through a graded ethanol series (96%, 80%, 70%). The sections were then stained with hematoxylin (BioVitrum, Russia) and eosin (DiaPath, Italy). After staining, the sections were dehydrated with ascending concentrations of ethanol (70%, 80%, 96%) and cleared in two washes of xylene, before being mounted with coverslips using Bio Mount synthetic medium (Bio Optica, Italy). Histological examination was performed using an Mshot microscope (MF52-N, China) at ×100 magnification, with photographs captured using an Mshot MS23 camera and analyzed with Mshot Image Analysis System software. Additional ×1,000 magnification with an oil immersion lens was used for further evaluation. A standardized calibration scale was used, and all measurements were recorded in μm. Pathological changes were assessed using a histological scoring system detailed in **Supplementary Table S1**.

### Animal housing and ethical considerations

All experiments involving laboratory animals were conducted at the vivarium of the M. Aikimbayev National Scientific Center for Especially Dangerous Infections (NSCEDI), Ministry of Health of the Republic of Kazakhstan. The housing, care, and feeding of SPF BALB/c mice were performed according to previously established protocols (22, 23). The study was conducted in compliance with Protocol #16, dated 31.10.2022, approved by the Institutional Animal Care and Use Committee (IACUC) at NSCEDI. All procedures adhered to both national and international regulations and guidelines governing the ethical treatment of laboratory animals. Mice were anesthetized via intraperitoneal (IP) injection of ketamine (50 mg/kg) and xylazine (10 mg/kg) in sterile phosphate-buffered saline. Humane endpoint criteria, as defined by IACUC-approved scoring parameters, were applied to determine when euthanasia was necessary. For terminal anesthesia before lung sample collection, mice were euthanized using an IP injection of ketamine (100 mg/kg) and xylazine (40 mg/kg), followed by cervical dislocation.

### Statistical analysis

GraphPad Prism 9.0 (GraphPad Software, San Diego, CA, USA) was used for data visualization and statistical analysis. Differences in antibody levels, ear swelling test results, airway responsiveness, and lung pathology between experimental groups were assessed using Tukey’s multiple comparisons test. The relationship between allergic bronchial asthma signs in mice induced by pollen extracts from different *Artemisia* species - considering the content and ratio of major allergens Art v 1 and Art v 3 - was analyzed using the Pearson multivariable correlation method. A P-value <0.05 was considered statistically significant. All error bars in the graphs represent the standard error of the mean (SEM).

## Results

### *A. vulgaris* pollen extract elicits the strongest IgE response in sensitized mice

**Figure 1:**
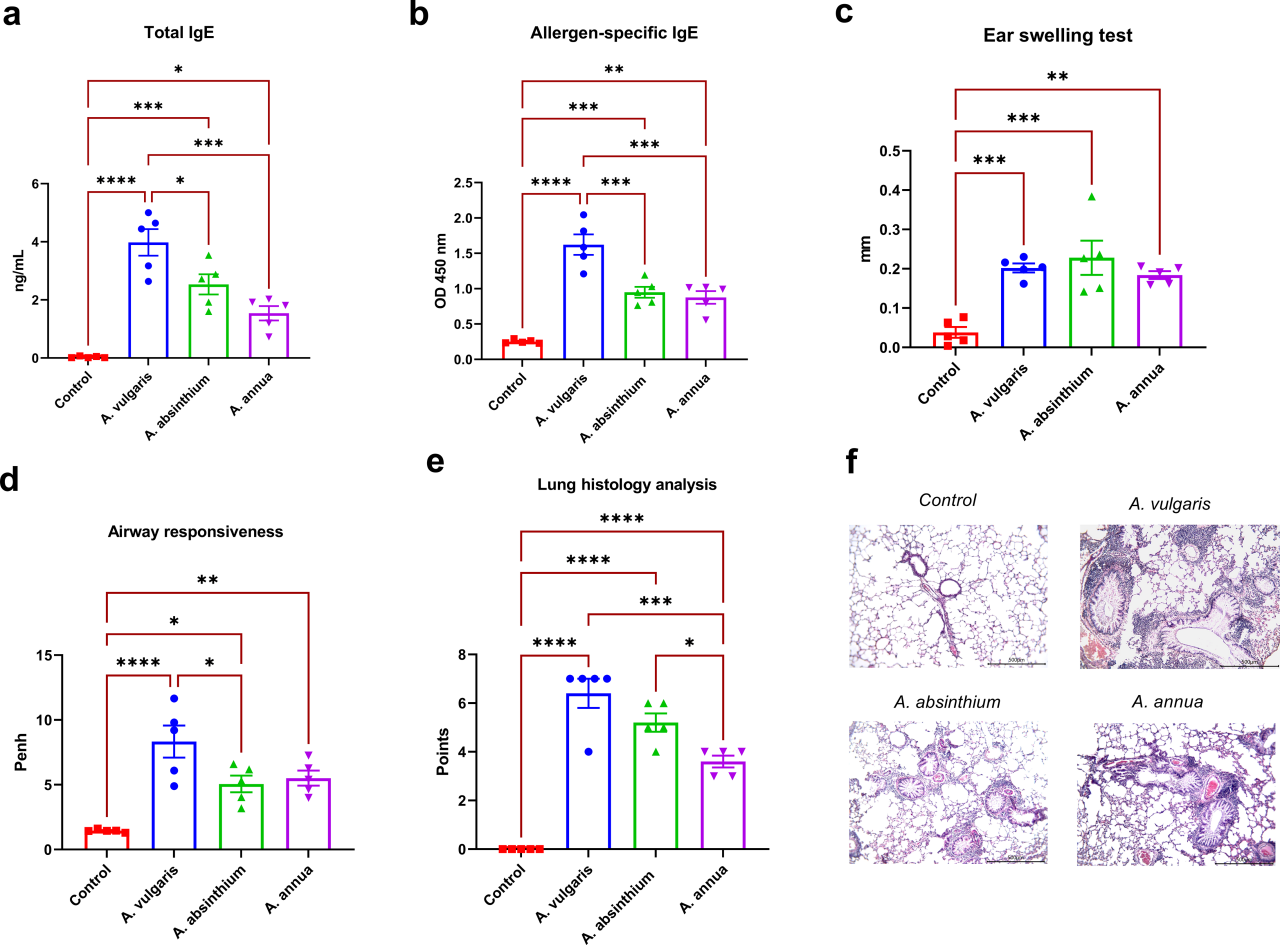
Comparative analysis of allergic responses to *A. vulgaris*, *A. absinthium*, and *A. annua* pollen extracts in a murine model. **(a)** Total IgE levels; the concentration of total IgE antibodies is expressed as ng/mL (serum diluted 1:200). **(b)** Allergen-specific IgE levels; allergen-specific IgE antibody levels are shown as optical density values at 450 nm (serum diluted 1:10). **(c)** Ear swelling test; results are presented as the delta in auricle thickness between allergen-injected and PBS-injected ears (expressed in mm). Measurement of ear thickness increase in mice following challenge with respective *Artemisia* pollen extracts; **(d)** Airway responsiveness; assessment of airway hyperreactivity to methacholine challenge using whole-body plethysmography; **(e)** Lung histology analysis; evaluation of peribronchial inflammation, eosinophil infiltration, and goblet cell metaplasia based on a 7-point scoring system. **(f)** overview lung pictures of mice in different groups, x100 magnification. Differences in the studied parameters between animal groups were assessed using Tukey’s multiple comparisons test. A P<0.05 value was considered a significant difference. *P<0.05, **P<0.01, ***P<0.001, and ****P<0.0001.

In BALB/c mice, sensitization followed by challenge with pollen extracts of *A. vulgaris*, *A. absinthium*, and *A. annua* resulted in a significant increase in both total and allergen-specific serum IgE levels compared to the control group (**Figures 1a, b**). The most pronounced elevation of these immunological parameters was observed in mice exposed to *A. vulgaris* pollen extract, which exhibited substantially higher IgE levels than those induced by other tested allergens. In contrast, sensitization with *A. absinthium* and *A. annua* extracts led to lower comparable total and allergen-specific IgE responses.

### *Artemisia* pollen extracts induce similar ear swelling responses

All tested *Artemisia* pollen extracts elicited a positive ear swelling response, a key laboratory marker of allergic inflammation, in sensitized BALB/c mice. This response was characterized by a significant increase in ear swelling compared to the control group following respective allergen administration (**Figure 1c**). No significant differences were observed in the degree of ear thickness increase among the extracts from different *Artemisia* species. These findings indicate a comparable allergenic potential of the tested pollen extracts within the applied experimental model.

### *A. vulgaris* elicits the highest airway hyperresponsiveness among three artemisia allergens

Twenty-four hours after the challenge with three *Artemisia* respiratory allergens, airway hyperreactivity was assessed in all experimental groups of mice using whole-body plethysmography in response to methacholine inhalation. All tested allergens induced a significant increase in airway reactivity compared to the control group (**Figure 1d**). Notably, the highest level of airway hyperresponsiveness was observed in mice sensitized to *A. vulgaris* pollen extract, while *A. absinthium* and *A. annua* elicited comparable responses.

### Pulmonary pathology severity: *A. vulgaris* dominant, *A. absinthium* intermediate, *A. annua* mild

Histological analysis of lung samples following allergen challenge was performed using a standardized scoring system that assessed perivascular and peribronchial inflammation, the presence of eosinophils in inflammatory foci, and goblet cell metaplasia in the bronchi. The analysis revealed significantly greater pulmonary pathology in sensitized mice challenged with *Artemisia* pollen extracts compared to the control group, which showed no lung abnormalities (**Figures 1e, f**; **Supplementary Table S2**). Among the tested allergens, *A. vulgaris* induced the most severe lung alterations, characterized by extensive lymphocytic peribronchial inflammation with numerous eosinophils (4/5 mice) and pronounced goblet cell metaplasia, resulting in the highest pathology scores (average: 6.4, max: 7). *A. absinthium* caused moderate peribronchial inflammation with scattered eosinophils and substantial goblet cell metaplasia, leading to intermediate pathology scores (average: 5.2). *A. annua* elicited the mildest changes, with focal peribronchial inflammation, occasional eosinophils, and minimal goblet cell metaplasia, reflected in the lowest pathology scores (average: 3.6). Notably, lung pathology in the *A. annua* group was significantly lower than in both *A. vulgaris*- and *A. absinthium*-sensitized mice.

### Art v 1 content and its dominant ratio to Art v 3 as key predictors of allergic inflammation and lung pathology in *Artemisia* pollen allergy

**Figure 2:**
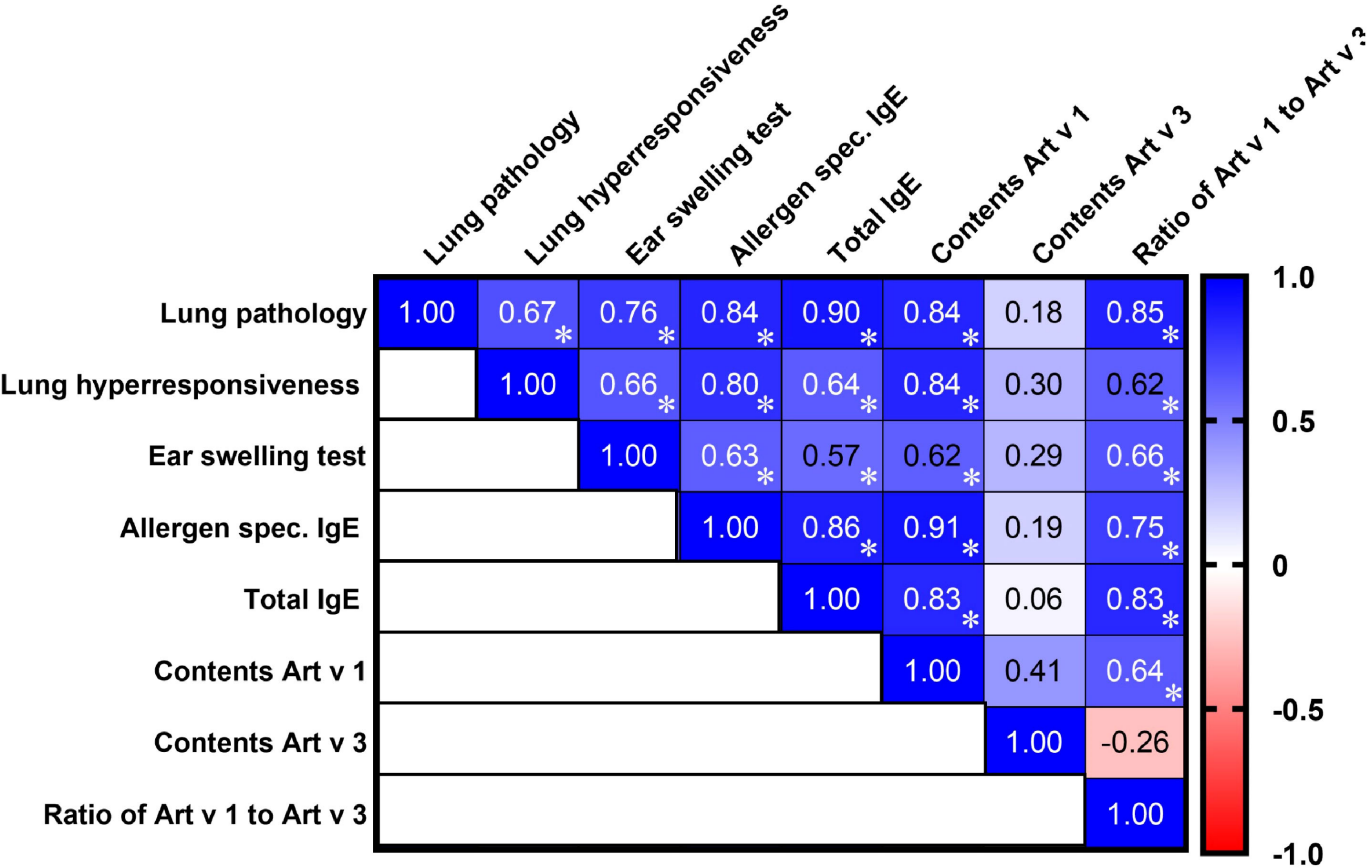
Correlation matrix analysis of allergic bronchial asthma signs in mice induced by *A. vulgaris*, *A. absinthium*, and *A. annua* pollen extracts, considering the content and ratio of the major allergens Art v 1 and Art v 3. The color refers to the r value scale (from -1 to 1) shown on the right. The number in each cell represents the actual r value. The analysis was performed using Pearson’s multivariate correlation method. Normalized data were used for this analysis. *P ≤ 0.05.

A multiple correlation analysis revealed that the levels of major allergenic proteins Art v 1 and Art v 3, as well as their ratio in *Artemisia* pollen extracts, exhibited significant associations with the severity of bronchial asthma and laboratory markers of allergic inflammation (**Figure 2**). A high Art v 1 content showed a strong positive correlation with the extent of pathological lung changes (*r* = 0.84), airway hyperreactivity (*r* = 0.84), total IgE levels (*r* = 0.83), and allergen-specific IgE levels (*r* = 0.91), while its correlation with ear swelling test results was moderate (*r* = 0.62). In contrast, Art v 3 levels in pollen extracts showed no significant correlation with pathological, clinical, or laboratory indicators of allergy in sensitized mice. Notably, the Art v 1/Art v 3 ratio demonstrated the highest positive correlation (*r* = 0.85) with the severity of lung pathology following allergen challenge, exceeding the correlation observed for each of these proteins individually.

Regarding the interrelationships among the studied clinical and laboratory markers of allergy, all correlations were positive, either strong or moderate. The strongest correlations were observed between lung pathology and total IgE (*r* = 0.90), allergen-specific IgE (*r* = 0.84), and ear swelling test results (*r* = 0.76), underscoring their close association in the mechanisms of allergic lung inflammation. Moderate positive correlations were observed between lung pathology and airway hyperreactivity (r = 0.67), which, in turn, showed a strong correlation with allergen-specific IgE (r = 0.80) and only moderate associations with the ear swelling test (r = 0.66) and total IgE levels (r = 0.64).

Thus, the Art v 1 allergen content in *Artemisia* pollen extracts strongly correlates with the severity of allergic inflammation, while Art v 3 shows no significant associations. The Art v 1/Art v 3 ratio is a stronger predictor of lung pathology than either protein alone, highlighting its potential role in modulating allergic responses.

## Discussion

This study provides the first comparative analysis of the allergenic potential of three *Artemisia* species (*A. vulgaris*, *A. absinthium*, and *A. annua*) using a BALB/c mouse model of bronchial asthma, assessed through clinical, pathological, and laboratory parameters. For accuracy, sensitization and allergen challenge of mice were performed using equal doses of extracts, standardized by total protein content. *A. vulgaris* pollen exhibited the highest allergenic properties, inducing the most significant immune and inflammatory responses, characterized by elevated IgE levels, airway hyperresponsiveness, and severe pulmonary pathology. While *A. absinthium* and *A. annua* showed comparable allergenic potential, *A. absinthium* induced significantly greater pulmonary pathological alterations.

An even more significant outcome of this study was our attempt to establish a relationship between clinical, pathological manifestations of bronchial asthma, and laboratory indicators of allergy and the content of major allergens Art v 1 and Art v 3 in pollen extracts from *A. vulgaris*, *A. absinthium*, and *A. annua*. Correlation analysis of major allergen levels strongly supports that Art v 1, rather than Art v 3, is the key determinant of *Artemisia* pollen allergenicity, which is consistent with multiple previous studies (17, 18, 20). This was clearly illustrated in *A. annua*, where Art v 3 levels were 3.5 to 8 times higher than in other species, yet its pulmonary inflammatory potential remained the lowest among the studied species of *Artemisia*. However, the most unexpected and significant finding was the strong correlation between the severity of pulmonary inflammatory responses and the Art v 1/Art v 3 ratio. Our data indicates that the predominant presence of Art v 1 over Art v 3 is the key factor determining the allergenic potential of *Artemisia* pollen in bronchial asthma laboratory models. These findings highlight the critical role of Art v 1, particularly its dominance over Art v 3, in driving respiratory allergic diseases, with bronchial asthma being the most severe manifestation. In contrast, although Art v 3 did not correlate with asthma-like pathology in our mouse model, it belongs to the nsLTP family of pan-allergens, which are known to be involved in cross-reactive allergic responses to plant-derived foods. Prior studies have shown that sensitization to Art v 3 can lead to IgE cross-reactivity with nsLTPs from peach, walnut, and celery, contributing to clinical syndromes such as mugwort-celery-spice syndrome, suggesting that Art v 3 may play a more prominent role in pollen–food cross-reactivity rather than respiratory pathology (24, 25).

It is important to emphasize that the levels of major allergens Art v 1 and Art v 3, as well as their relative proportions in *Artemisia* pollen, can vary significantly depending on geographic location and climatic conditions, even within the species analyzed in this study. For instance, according to Zhao et al. (20), Art v 3 was predominant over Art v 1 in *A. vulgaris* pollen extracts, whereas Art v 1 was more abundant than Art v 3 in *A. annua*. Given these findings, the allergenicity ranking established for *A. vulgaris*, *A. absinthium*, and *A. annua* in our study should currently be considered specific to their geographically distinct groups in Kazakhstan. This underscores the need for further investigations to assess how ecological factors influence the allergenic composition of *Artemisia* pollen. To further support our hypothesis, future studies will investigate the allergenic potential of additional *Artemisia* species, particularly the Chinese endemics *A. annua*, *A. argyi*, and *A. sieversiana* (26). As in Kazakhstan, *Artemisia* pollen is a dominant airborne allergen as in China (27, 28). Moreover, future research should also aim to compare pollen samples from different geographical regions and, where possible, correlate the Art v 1/Art v 3 ratio or other important allergen components with clinical manifestations and sensitization patterns in allergic patients across various populations. Such approaches would strengthen the translational value of our findings and support the development of regionally tailored standardization of diagnostic and therapeutic strategies.

These findings confirm a strong positive correlation between the severity of bronchial asthma manifestations in mice, following challenge with pollen extracts from *A. vulgaris, A. absinthium*, and *A. annua*, and key laboratory markers of allergy, including total and allergen-specific IgE, as well as the ear swelling test. The strongest associations were observed between lung pathology and IgE levels, underscoring the central role of IgE-mediated sensitization in allergic airway inflammation. Moderate correlations between lung pathology and airway hyperreactivity, along with its strong link to allergen-specific IgE, further highlight the IgE-driven mechanisms of pollen-induced asthma. While the ear swelling test showed moderate associations with airway hyperreactivity and total IgE, it remains a valuable marker of local allergic inflammation. These results reinforce the importance of IgE and ear swelling responses as indicators of allergenic potential and warrant further investigation into the influence of pollen composition on immune reactions.

The present study highlights the dominant role of Art v 1 in *Artemisia*-induced allergy, including its most severe manifestation - bronchial asthma. Our findings are in agreement with our previous research (22, 23, 29) and further support the development of an effective monocomponent vaccine based solely on recombinant Art v 1 for both subcutaneous and sublingual allergen-specific immunotherapy.

Although this study was conducted in a laboratory mouse model and its findings cannot be directly applied to humans, it addresses a key gap in clinical practice, where there is no objective method for determining individual allergenicity to specific *Artemisia* species. Our data offers valuable insights into identifying the most asthma-triggering *Artemisia* species, considering variations in the levels and ratios of the major allergens Art v 1 and Art v 3. Nevertheless, caution is warranted when translating these findings to human allergic responses, as there are inherent differences between murine and human immune systems that may influence allergen recognition and response. Additionally, the exposure conditions in experimental models differ from real-life environmental exposure routes and patterns, which could further affect clinical relevance. A limitation of our standardization approach based on total protein content is the potential variability in the composition of other allergenic or immunomodulatory proteins across the *Artemisia* species. While our study focused on Art v 1 and Art v 3, SDS-PAGE analysis revealed differences in overall protein profiles, highlighting the possibility that additional extract components could contribute to the observed allergenicity. Although our findings show a strong correlation between Art v 1 content and allergic outcomes, it is important to note that whole pollen extracts may contain additional allergens beyond Art v 1 and Art v 3. Therefore, the observed IgE reactivity should be interpreted as reflecting source-specific rather than strictly allergen-specific sensitization. Future studies using purified or recombinant allergens will be necessary to confirm the individual contributions of specific components. Due to the pilot nature of the investigation and resource constraints, we did not perform sequencing of Art v 1 and Art v 3 genes or proteins across the three *Artemisia* species. While such data would provide additional molecular insight, previous studies (19) have demonstrated that Art v 1 is highly conserved among *Artemisia* species (>95% amino acid identity), particularly within its defensin-like domain, suggesting minimal interspecies variability in this major allergen. In contrast, Art v 3 is more polymorphic only between different sections within *Atremisia* genus (20), so we expect *A. absinthium* has quite similar sequences to *A. sieversiana* because their close relationship in phylogenomic tree (5). However, in our model, it did not correlate with key asthma-related outcomes. Therefore, sequence analysis remains a worthwhile objective for future research. A limitation of this study is the absence of IgE immunoblotting, which would have allowed visualization of the molecular weight and IgE reactivity of individual allergens in the extracts. This was due to the unavailability of specific antibodies or patient sera at the time. Future studies incorporating immunoblot analysis are warranted to further validate the allergen content and composition of Artemisia extracts.

## Conclusion

This study provides a comparative analysis of the allergenic potential of *A. vulgaris, A. absinthium*, and *A. annua* in a BALB/c mouse model of bronchial asthma. *A. vulgaris* was identified as the most potent allergen, inducing the highest IgE response, airway hyperreactivity, and severe pulmonary pathology, while *A. absinthium* had intermediate allergenicity and *A. annua* the mildest. A key finding is that Art v 1, rather than Art v 3, plays a dominant role in *Artemisia*-induced allergic inflammation, showing a strong correlation with key asthma markers. Notably, the Art v 1/Art v 3 ratio emerged as an even stronger predictor of lung pathology, emphasizing its potential role in modulating allergic responses. Although Art v 1 and its ratio to Art v 3 correlate strongly with allergic outcomes in our model, these conclusions are based on whole-extract sensitization, and the presence of other allergens cannot be ruled out.

Future studies should explore other *Artemisia* species, especially those endemic to China, where *Artemisia* pollen is a major airborne allergen. Identifying ecological and molecular factors in *Artemisia*-induced allergy will help optimize diagnostic and therapeutic strategies for allergic diseases, particularly bronchial asthma.

## Data Availability

The original contributions presented in the study are included in the article/**Supplementary Material**. Further inquiries can be directed to the corresponding author.
