# Supplementary figures and images for: The role of major allergens Art v 1 and Art v 3 in Artemisia pollen-induced asthma: a mouse model study
Source: Front Immunol. 2025 Jun 3;16:1590791. doi: 10.3389/fimmu.2025.1590791 (PMC12170315; doi:10.3389/fimmu.2025.1590791)

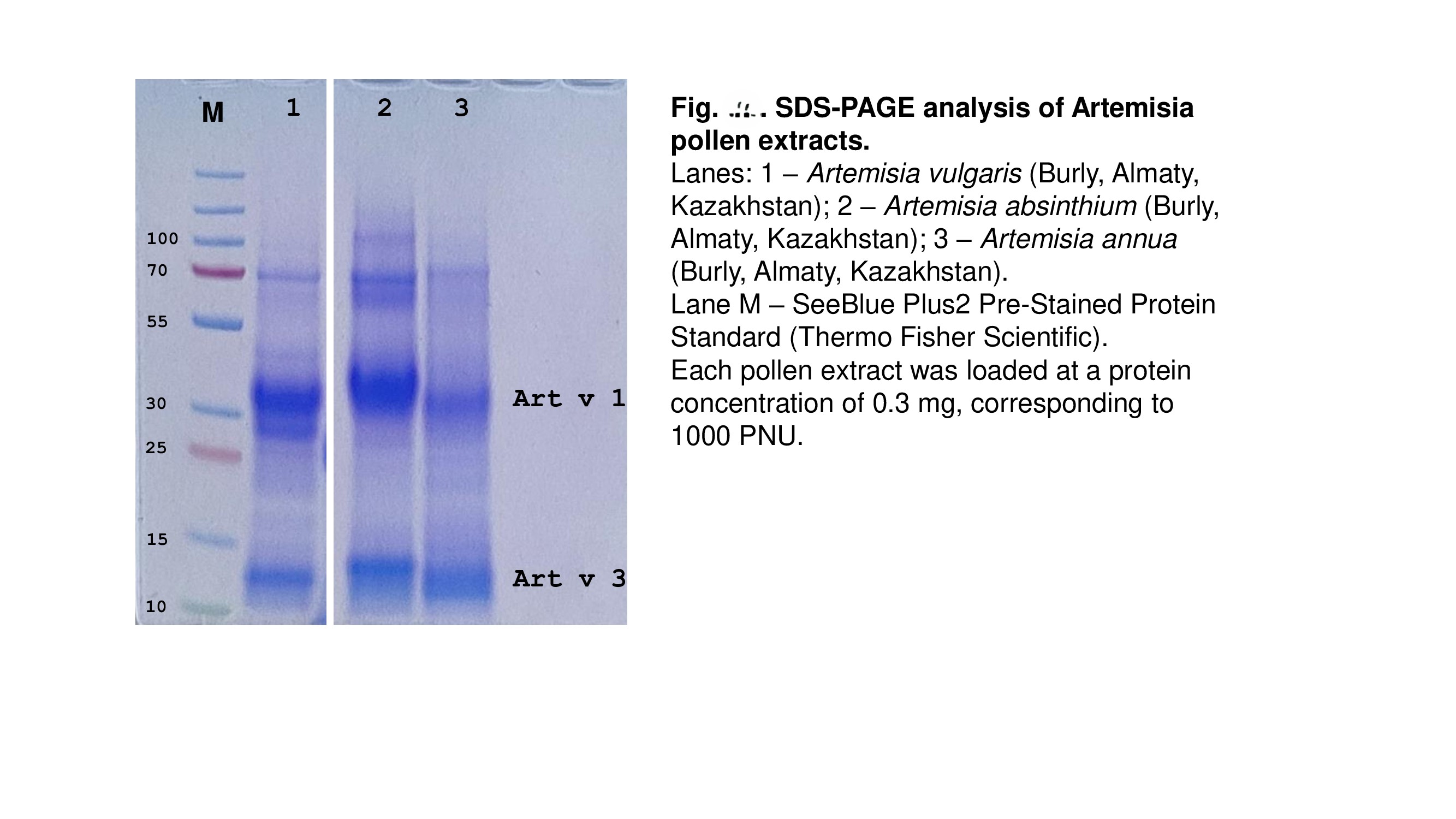

Supplement: Supplementary file 1 [file Image1.jpeg]

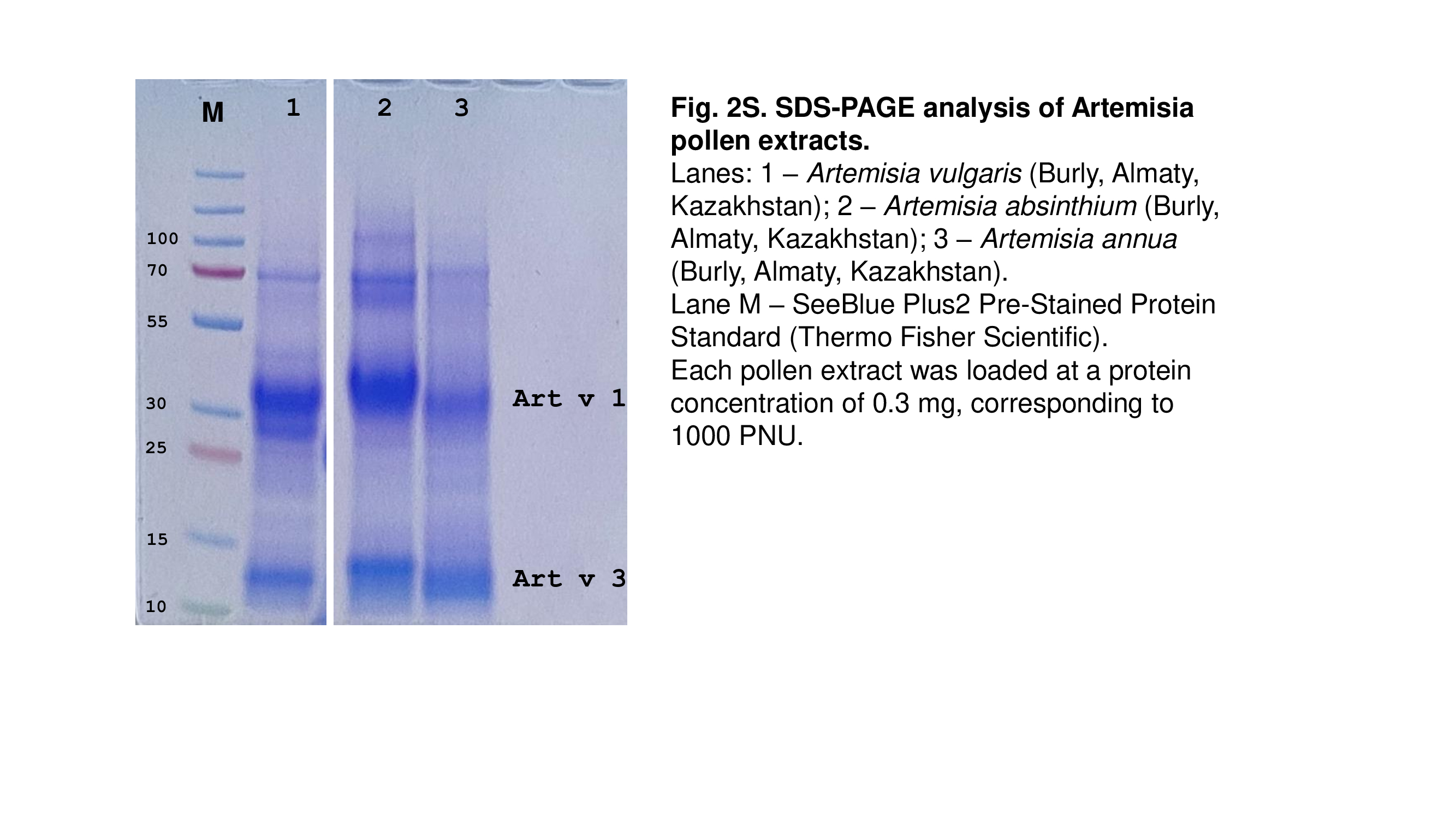

Supplement: Supplementary file 4 [file SupplementaryFile1.jpeg]
