# Supplementary material for: The role of major allergens Art v 1 and Art v 3 in Artemisia pollen-induced asthma: a mouse model study
Source: Front Immunol. 2025 Jun 3;16:1590791. doi: 10.3389/fimmu.2025.1590791 (PMC12170315; doi:10.3389/fimmu.2025.1590791)
Supplement: Supplementary file 2 [file Table1.docx]

**Supplementary Table S1.** Scoring System for Evaluating Pathological Changes in Mouse Lungs

| Evaluated trait | Points for the evaluated trait |
| --- | --- |
| Perivascular/peribronchial inflammation | 0 – no changes;  1 – moderate inflammation;  2 – pronounced inflammation;  3 – severe inflammation |
| Presence of eosinophils in foci of perivascular/peribronchial inflammation | 0 – absent;  1 – single eosinophils in the field with magnification (x1000);  2 – multiple eosinophils in the field with magnification (x1000) |
| Metaplasia of the Goblet cells in the bronchi | 0 – absent;  1 – several Goblet cells are present in one or two bronchiolar profiles;  2 – numerous Goblet cells are present in bronchioles |
| Maximum score | 7 |
