# Supplementary material for: The role of major allergens Art v 1 and Art v 3 in Artemisia pollen-induced asthma: a mouse model study
Source: Front Immunol. 2025 Jun 3;16:1590791. doi: 10.3389/fimmu.2025.1590791 (PMC12170315; doi:10.3389/fimmu.2025.1590791)
Supplement: Supplementary file 3 [file Table2.docx]

**Supplementary Table S2. Evaluation of Pathological Changes in Mouse Lungs** (Data Presented Per Individual Animal)

| Mouse Number | Evaluated trait | | | Cumulative Score |
| --- | --- | --- | --- | --- |
|  | *Perivascular/peribronchial inflammation* | *Presence of eosinophils in foci of perivascular/peribronchial inflammation* | *Metaplasia of the Goblet cells in the bronchi* |  |
| Control Group | | | | |
| 1 | 0 | 0 | 0 | 0 |
| 2 | 0 | 0 | 0 | 0 |
| 3 | 0 | 0 | 0 | 0 |
| 4 | 0 | 0 | 0 | 0 |
| 5 | 0 | 0 | 0 | 0 |
| *A. vulgaris* Group | | | | |
| 1 | 3 | 2 | 2 | 7 |
| 2 | 3 | 2 | 2 | 7 |
| 3 | 2 | 1 | 1 | 4 |
| 4 | 3 | 2 | 2 | 7 |
| 5 | 3 | 2 | 2 | 7 |
| *A. absinthium* Group | | | | |
| 1 | 2 | 2 | 2 | 6 |
| 2 | 2 | 1 | 1 | 4 |
| 3 | 2 | 1 | 2 | 5 |
| 4 | 2 | 1 | 2 | 5 |
| 5 | 2 | 2 | 2 | 6 |
| *A. annua* Group | | | | |
| 1 | 2 | 1 | 1 | 4 |
| 2 | 2 | 1 | 1 | 4 |
| 3 | 2 | 1 | 1 | 4 |
| 4 | 1 | 1 | 1 | 3 |
| 5 | 1 | 1 | 1 | 3 |
